# Dimethyl sulfoxide perturbs sleep-related behavioral organization in multiple species and confounds interpretation of sleep phenotypes

**DOI:** 10.3389/ftox.2026.1907359

**Published:** 2026-08-21

**Authors:** Nanami Udo, Manabu Sekiguchi, Etsuko Sawatari, Taichi Q. Itoh

**Affiliations:** 1 Graduate School of Systems Life Sciences, Kyushu University, Fukuoka, Japan; 2 Faculty of Arts and Science, Kyushu University, Fukuoka, Japan

**Keywords:** behavioral phenotyping, DMSO, *Drosophila melanogaster*, *Hydra vulgaris*, sleep architecture, sleep fragmentation, toxicophysiology

## Abstract

**Introduction:**

Dimethyl sulfoxide (DMSO) is a very commonly used solvent in biological research and generally considered an inert experimental vehicle. However, DMSO can alter physiological functions, raising concerns regarding its effects on behavioral phenotypes. Such concerns may be particularly important in sleep research because sleep-related behaviors are highly sensitive to physiological perturbations.

**Methods:**

In this study, we systematically examined the effects of DMSO on sleep-related behaviors in *Drosophila melanogaster* and *Hydra vulgaris*.

**Results:**

In *Drosophila*, DMSO dose-dependently altered nighttime sleep, increased locomotor activity, prolonged sleep latency, reduced average sleep-bout length, and increased sleep-bout number. In *Hydra*, 2% DMSO increased total sleep-like behavior together with an increased number of sleep bouts while reducing waking locomotor activity and sleep latency. Although the direction of total sleep changes differed between these species, both organisms exhibited disrupted sleep-related behavioral organization following DMSO exposure. Furthermore, high concentrations of DMSO induced severe physiological impairment, including a shortened lifespan in *Drosophila* and morphological deterioration and death in *Hydra*.

**Discussion:**

These findings demonstrate that DMSO is not merely a passive solvent in sleep research, but a biologically active perturbant, which is capable of confounding sleep-related behavioral phenotypes in both *Drosophila* and *Hydra*. Our study highlights the importance of carefully evaluating solvent effects in behavioral and pharmacological studies of sleep.

## 1 Introduction

Sleep is a fundamental and evolutionarily conserved behavior observed in diverse animal species (Rattenborg and Ungurean, 2023). Despite decades of research, the molecular and physiological mechanisms underlying sleep regulation remain poorly understood. Sleep is not controlled by a single pathway but emerges from complex integration of multiple physiological processes including neuronal activity, metabolism, oxidative stress, membrane excitability, circadian timing, and homeostatic regulation (Saper et al., 2010; Sehgal and Mignot, 2011; Joiner, 2016). Because of this complexity, sleep architecture is highly sensitive to physiological perturbations, making the interpretation of sleep-related phenotypes particularly challenging.

Pharmacological approaches are widely used in sleep research to investigate the molecular mechanisms underlying sleep and arousal (Rihel et al., 2010; Nall and Sehgal, 2013; Rihel and Schier, 2013). In these studies, hydrophobic compounds are commonly dissolved in vehicle solvents prior to administration. Dimethyl sulfoxide (DMSO) is a highly frequently used solvent owing to its high solubilizing capacity and membrane permeability (Santos et al., 2003; Gurtovenko and Anwar, 2007). Importantly, DMSO is generally treated as an inert experimental vehicle, and its low concentrations are often assumed to have minimal biological impact.

However, DMSO is not biologically inert. It alters membrane permeability, lipid organization, and fluidity, thereby influencing various cellular processes (Lyman et al., 1976; Santos et al., 2003; Notman et al., 2006; Gurtovenko and Anwar, 2007). Even, concentrations commonly regarded as “safe” (0.1%–1%) have been reported to induce measurable physiological effects in multiple systems (Tunçer et al., 2018). In invertebrate models, DMSO has been associated with developmental delays, altered locomotor activity, and neuronal dysfunction, whereas recent studies on vertebrates have demonstrated concentration-dependent morphological and physiological abnormalities (AlOkda and Van Raamsdonk, 2022; Gomes et al., 2025; Thi Thu Trinh et al., 2025). These findings raise concerns that DMSO itself may perturb behavioral phenotypes, thereby confounding the interpretation of pharmacological studies.

These concerns are particularly important for sleep research. Because sleep is highly susceptible to subtle physiological disturbances, solvent-induced changes in neuronal excitability, oxidative stress, membrane properties, or systemic physiology may be misinterpreted as sleep-specific phenotypes. Indeed, DMSO can alter sleep architecture in rodents (Cavas et al., 2005). However, the extent, to which DMSO itself affects sleep behavior and architecture in *Drosophila* and *Hydra* remains poorly understood.

In this study, we systematically analyzed the effects of DMSO on sleep and behavior in two evolutionarily distant model organisms, *Drosophila melanogaster* and *Hydra vulgaris*. We assessed the effects of DMSO on sleep architecture, locomotor activity, and physiological integrity to examine whether it is a biologically active perturbant capable of confounding behavioral phenotypes.

## 2 Materials and methods

### 2.1 Fly strains and rearing

*Drosophila melanogaster w* ^*1118*^ (BDSC #5905) was used in this study. Flies were reared at 25 °C under a 12-h/12-h light–dark (LD12:12) cycle on *Drosophila* medium comprising 0.8% agar, 10% glucose, 5% cornmeal, 4% dry yeast, 3.2% wheat germ, 1.2% Bokinin, and 0.5% propionic acid (all w/v or v/v).

### 2.2 Hydra strains and rearing

Non-budding *H*. *vulgaris* (strain 105) was used for all experiments. Animals were maintained in Hydra culture solution (HCS: 1 mM NaCl, 1 mM CaCl_2_, 0.1 mM KCl, 0.1 mM MgSO_4_, and 1 mM Tris-[hydroxymethyl]-aminomethane), adjusted to pH 7.4 with HCl. All cultures were kept at 20 °C under a LD12:12 cycle (light intensity, 450 lx). Animals were fed twice weekly with freshly hatched *Artemia nauplii*, and the culture medium was replaced the following day. Prior to locomotor-activity measurements, animals were fasted for 24 h.

### 2.3 Behavioral recording of *Drosophila* and data analysis

Behavioral recordings were conducted using young (8–9 days post-eclosion) and aged (35–37 days post-eclosion) male and female flies. Individual flies were anesthetized with triethylamine and placed in recording tubes containing standard food comprising 2% agar and 4% sucrose with different concentrations of DMSO (0.05%, 0.5%, 1%, and 2%). Locomotor activity was monitored using a *Drosophila* Activity Monitor system (DAM2; TriKinetics Inc., Waltham, MA, USA). All experiments were conducted within a temperature-controlled incubator (FMU-054I; Fukushima Galilei, Osaka, Japan) maintained at 25 °C. Illumination was provided by white light-emitting diodes positioned above the monitors and regulated by a digital timer (PT70DW; REVEX Co., Ltd., Saitama, Japan). Light intensity was maintained at 500 lx throughout the experiment. All experimental conditions, including both young and aged cohorts, were tested in two independent experimental runs. Data from the independent runs were pooled for statistical analysis, and the sample sizes (n) shown in the figures represent the total number of flies included in the pooled analysis. Flies showing complete cessation of locomotor activity by the end of the recording period were considered non-viable and excluded from the behavioral analyses. Accordingly, only flies that remained active at the end of the recording period were included in the final analyses. Sleep and behavioral analyses were performed using custom-written Python scripts. Sleep-related immobility was defined as a period of inactivity lasting ≥5 min, consistent with the established criteria previously described (Hendricks et al., 2000; Shaw et al., 2000). Sleep bouts were defined as discrete sleep episodes separated by wake periods, and were quantified as the number of sleep bouts occurring within each 12-h light or dark phase. Average sleep-bout length (ABL) was calculated as the mean duration of individual sleep episodes within each phase. Sleep latency was defined as the time elapsed from the light–dark or dark–light transition to the onset of the first sleep episode. The total locomotor activity was calculated based on the number of infrared beam crossings, providing a measure of overall movement independent of sleep classification. In addition, waking activity was calculated by dividing the total number of infrared beam crossings by the total wake time and was expressed as beam crossings per waking minute. To minimize the influence of the sleep transition immediately after lights-off, waking activity during the dark phase was quantified using data collected during ZT13–24, excluding the first hour after lights-off (ZT12–13).

### 2.4 Behavioral recording of *Hydra* and data analysis

Prior to behavioral recording, animals were subjected to a 24-h starvation period. Each individual non-budding animal was transferred to a silicone chamber (16 mm × 16 mm × 5 mm) containing 1 mL HCS supplemented with DMSO at the specified concentrations. These chambers were placed within an incubator (CN-40A incubator; Mitsubishi Electric, Tokyo, Japan) at Zeitgeber time 10 (ZT10; where ZT0 and ZT12 indicate light-on and light-off, respectively). To ensure sufficient adaptation to the experimental environment, behavioral and sleep-related analyses were conducted beginning at ZT0 on the following day. To continuously monitor the behavior throughout light and dark phases, individuals were illuminated with 850-nm infrared light (Session Digital, Tokyo, Japan) and imaged using an E3 CMOS camera (Visualix, Hyogo, Japan) equipped with an infrared high-pass filter (FUJIFILM, Tokyo, Japan). The LD12:12 cycle was maintained with a white light-emitting diode at an intensity of 450 lx using a digital timer (PT70DW; REVEX Co., Ltd., Saitama, Japan). Images were captured at 5-s intervals at a resolution of 1,920 × 1,200 pixels and stored as 8-bit grayscale frames. Locomotor activity was quantified using a custom ImageJ macro based on frame-subtraction analysis as previously described (Kanaya et al., 2019). Sleep-related behavioral parameters were quantified as previously described (Kanaya et al., 2020; Sato et al., 2025). Individuals were excluded based on predefined activity thresholds (FOM < 0.1 or > 0.9), where FOM represents the mean fraction of movement calculated over 2-min intervals. These criteria were applied to remove individuals exhibiting extremely low or high overall activity across recording days.

### 2.5 Two-choice preference assay

The two-choice preference assay was performed as previously described (Tanimura et al., 1982), with modifications. To exclude the influence of age-related changes on food preference, experiments were conducted independently using young (3–8 days post-eclosion) and aged (30–35 days post-eclosion) flies. Approximately 40 flies per trial were fasted for 16 h prior to the assay. Without anesthesia, flies were transferred to a plastic petri dish (90 mm × 15 mm) and allowed to feed for 1 h at 25 °C under constant darkness. Two filter papers cut into squares (20 mm × 20 mm) and each soaked with a different sugar solution were symmetrically placed at equal distances from the center. One solution comprised a 5% sucrose base containing DMSO colored with blue food dye (0.125 mg/mL Brilliant Blue FCF). The other was a 5% sucrose solution colored with a red food dye (0.25 mg/mL Acid Red). Following the feeding period, flies were frozen at −30 °C for at least 3 h. The color of their abdomen was examined using a microscope and categorized into four groups: blue (B), red (R), purple (M), or colorless (O). Preference Index (PI) was calculated using the following (Equation 1):

$$\text{PI}=\left(B+M/2\right)/\;\left(B+M+R\right).$$ (1)

A PI of 1 indicated total preference for the blue DMSO-containing solution, whereas 0 indicated a total preference for the red control solution. Preference for DMSO was evaluated by statistically comparing the PI values between groups.

### 2.6 Survival assay under sucrose/agar conditions

For the survival assay, flies were used at 5–7 days post-eclosion. Under triethylamine anesthesia, flies were transferred to vials containing 10 flies each and the same medium used for the behavioral assays (4% sucrose and 2% agar). Each experimental condition consisted of five independent vials (10 flies per vial; 50 flies per condition). Flies were maintained under standard conditions, and the number of dead individuals was recorded two to three times per week during medium changes. Monitoring continued until all flies had died, and Kaplan–Meier survival curves were generated from the collected data.

### 2.7 Statistical analysis

Statistical analyses were performed using R v.4.3.3. For two-group comparisons, normality and homogeneity of variance were assessed using the Shapiro–Wilk and Brown–Forsythe tests, respectively. For normally distributed data, two-tailed Student’s or Welch’s *t*-test was used. For non-normally distributed data, the Mann–Whitney U test was used. When multiple pairwise two-group comparisons were conducted, *P*-values were adjusted for multiple testing using the Holm correction. For multiple-group comparisons, normality and homogeneity of variance were similarly tested. Depending on the data distribution and variance homogeneity, we performed one-way analysis of variance (ANOVA) followed by Tukey’s honestly significant difference (HSD) test or pairwise t-tests with Holm adjustment, Welch’s ANOVA followed by Dunnett’s T3 test or Games-Howell tests, the Kruskal-Wallis test followed by Dunn’s *post hoc* test with Holm or Hochberg adjustment, or Aligned Ranks Transformation ANOVA followed by Wilcoxon rank-sum tests with Holm adjustment. Survival data were analyzed using Kaplan–Meier survival curves, and differences between groups were assessed using the log-rank test. Statistical significance was set at *P* < 0.05.

## 3 Results

### 3.1 DMSO alters sleep-related behavioral parameters independently of feeding avoidance

To determine whether DMSO itself influenced sleep-related behaviors, we first performed a two-choice preference assay to exclude the possibility that the behavioral changes were secondary to feeding avoidance. Most experimental conditions showed no significant aversion to DMSO-containing food regardless of age, whereas young females showed significant avoidance only at the highest concentration tested (2% DMSO) (Supplementary Figures S1A,B).

**FIGURE 1:**
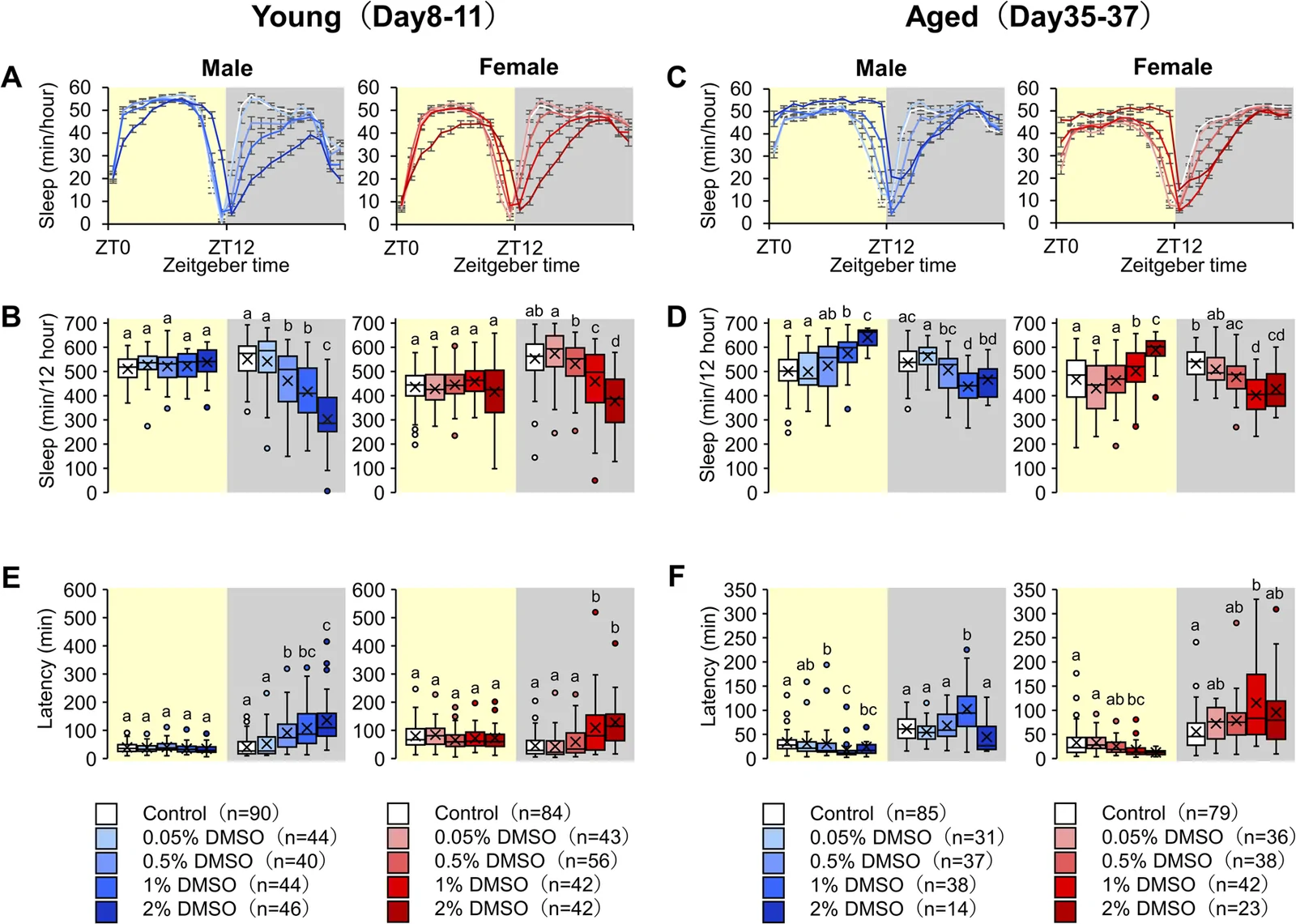
Dose-dependent effects of dietary DMSO on sleep-related behavioral parameters in *Drosophila*. **(A)** Mean daily sleep profiles of young flies under LD12:12 conditions. **(B)** Box plots representing total sleep amount of young flies during the light and dark phases. **(C)** Mean daily sleep profiles of aged flies under LD12:12 conditions. **(D)** Box plots representing total sleep amount of aged flies during the light and dark phases. **(E,F)** Sleep latency in young **(E)** and aged **(F)** flies. Male (blue) and female (red) flies are shown separately. Light phases (yellow) and dark phases (gray) are indicated. Different color intensities denote increasing concentrations of dietary DMSO. Sample sizes (n) are indicated in the figure. Statistical analyses were performed as follows: Kruskal–Wallis test for (**(B)** male light) and (**(E)** male and female light); Kruskal–Wallis test followed by Dunn’s multiple-comparison test with Hochberg adjustment for (**(D)** female light) and (**(F)** male light); one-way ANOVA followed by pairwise t-tests with Holm adjustment for (**(D)** male and female dark); aligned rank transform (ART) ANOVA for (**(B)** female light); and aligned rank transform (ART) ANOVA followed by pairwise Wilcoxon exact tests with Holm adjustment for (**(B)** male dark), (**(B)** female dark), (**(D)** male light), (**(E)** male dark), (**(E)** female dark), and (**(F)** male and female light and dark). Exact overall *P* values and adjusted *P* values are provided in Supplementary Table S2. Statistical significance was defined as *P* < 0.05. Groups sharing the same compact letter display (CLD) letter are not significantly different, whereas groups with different letters differ significantly.

Next, we examined the effects of DMSO on sleep-related behavioral parameters in young and aged flies of both sexes. During the light phase, DMSO had relatively limited effects on sleep amount in young flies. In contrast, during the dark phase, DMSO significantly altered sleep-related behaviors in a dose-dependent manner, particularly at concentrations between 0.5% and 2% (Figures 1A–D). These effects were observed across both sexes and age groups although the magnitude and concentration sensitivity varied among conditions.

We further analyzed sleep latency as an indicator of stability of sleep initiation. While latency after light-on was largely unaffected in young flies, DMSO significantly prolonged sleep latency after light-off in multiple experimental groups (Figures 1E,F). These findings indicate that DMSO perturbs multiple sleep-related behavioral parameters in *Drosophila*, independently of major feeding-avoidance effects.

### 3.2 DMSO disrupts sleep architecture in *Drosophila*

To evaluate whether DMSO affected sleep architecture, we analyzed the average sleep-bout length (ABL) and sleep-bout number.

**FIGURE 2:**
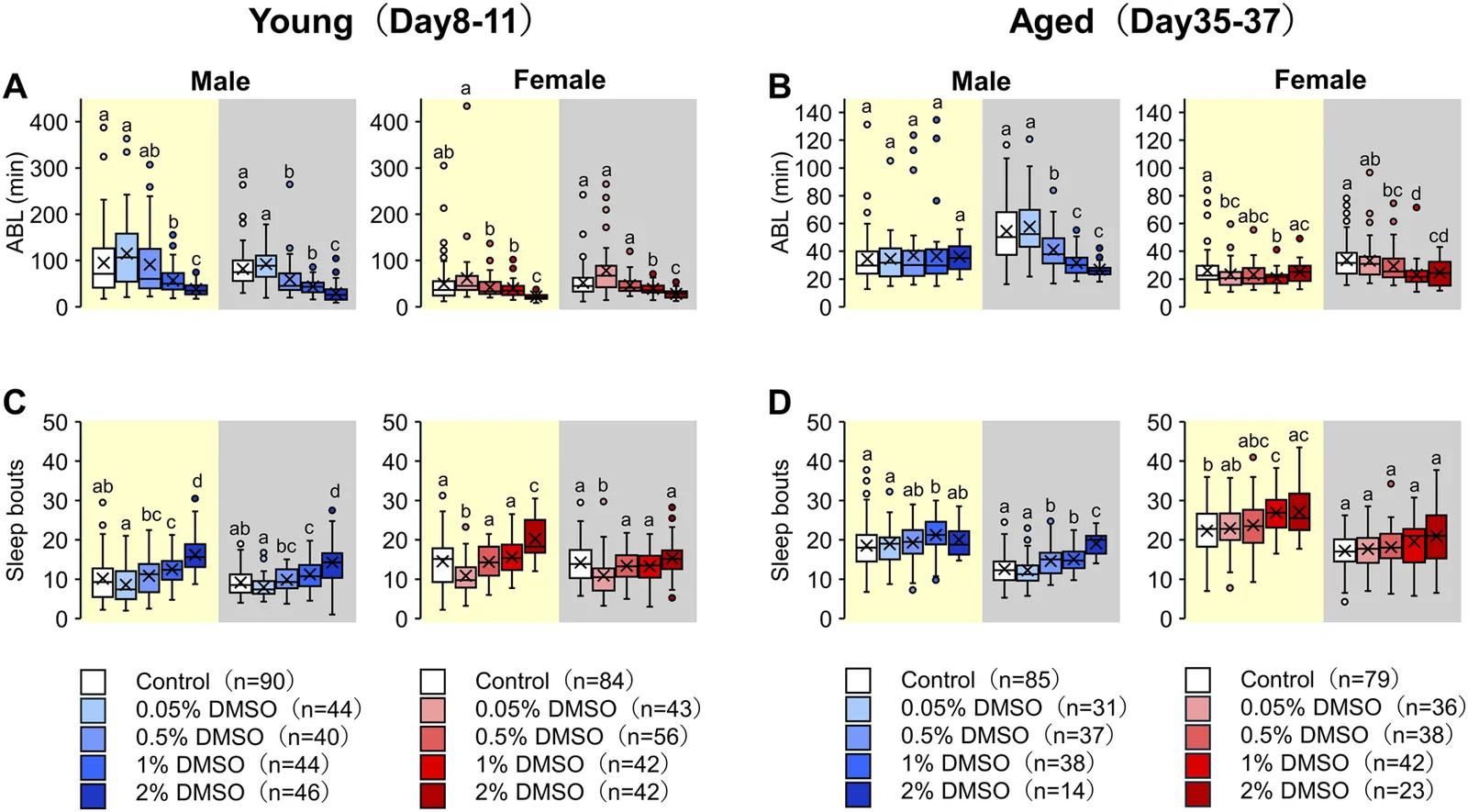
Effects of dietary DMSO on sleep architecture in *Drosophila*. **(A,B)** Box plots representing ABL in young and aged flies. **(C,D)** Box plots representing sleep-bout number in young and aged flies. Male (blue) and female (red) flies are shown separately. Light phases (yellow) and dark phases (gray) are indicated. Different color intensities denote increasing concentrations of dietary DMSO. Sample sizes (n) are indicated in the figure. Statistical analyses were performed as follows: aligned rank transform (ART) ANOVA followed by pairwise Wilcoxon exact tests with Holm adjustment for (**(A)** male and female light and dark), (**(B)** male dark), (**(C)** male dark), and (**(C)** female light); Kruskal–Wallis test for (**(B)** male and female light), followed by Dunn’s multiple-comparison test with Hochberg adjustment for (B, female dark), (**(C)** male light), (**(C)** female dark), and (**(D)** male light and dark); one-way ANOVA followed by pairwise t-tests with Holm adjustment for (**(D)** female light); and Welch ANOVA for (**(D)** female dark). Exact overall *P* values and adjusted *P* values are provided in Supplementary Table S3. Statistical significance was defined as *P* < 0.05. Groups sharing the same compact letter display (CLD) letter are not significantly different, whereas groups with different letters are significantly different.

DMSO reduced ABL, particularly during the dark phase (Figures 2A,B). In parallel, DMSO dose-dependently increased the number of sleep bouts (Figures 2C,D). These effects were consistently observed across multiple sex and age groups, although the precise concentration sensitivity differed among conditions.

Importantly, the simultaneous reduction in ABL and increase in sleep-bout number indicate fragmentation of sleep architecture rather than a simple increase or decrease in total sleep duration. These findings suggest that DMSO destabilizes sleep architecture in *Drosophila*.

### 3.3 DMSO dose-dependently alters locomotor activity

**FIGURE 3:**
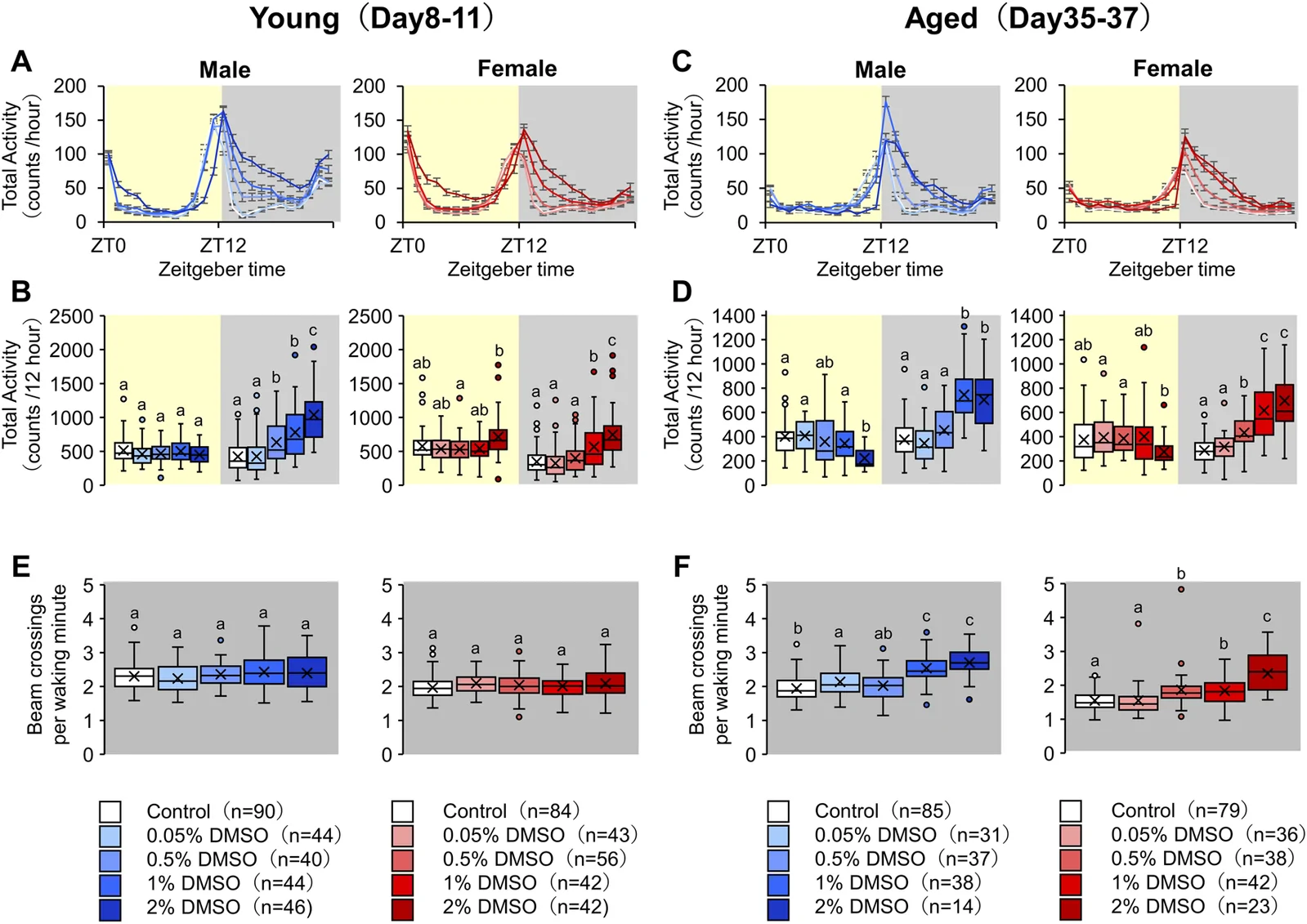
Dose-dependent effects of dietary DMSO on locomotor activity in *Drosophila*. **(A)** Mean daily locomotor activity profiles of young flies under LD12:12 conditions. **(B)** Box plots representing total locomotor activity in young flies during light and dark phases. **(C)** Mean daily locomotor activity profiles of aged flies under LD12:12 conditions. **(D)** Box plots representing total locomotor activity in aged flies during light and dark phases. **(E)** Beam crossings per waking minute during the dark phases (ZT13–ZT24) in young flies. **(F)** Beam crossings per waking minute during the dark phases (ZT13–ZT24) in aged flies. Male (blue) and female (red) flies are shown separately. Light phases (yellow) and dark phases (gray) are indicated. Sample sizes (n) are indicated in the figure. Statistical analyses were performed as follows: Kruskal–Wallis test for (**(B)** male light) and (**(E)** female); aligned rank transform (ART) ANOVA for (**(E)** male); aligned rank transform (ART) ANOVA followed by pairwise Wilcoxon exact tests with Holm adjustment for (**(B)** male dark), (**(B)** female light and dark), (**(D)** male light and dark), (**(D)** female light and dark), and (**(F)** female); and Kruskal–Wallis test followed by Dunn’s multiple-comparison test with Hochberg adjustment for (**(F)** male). Exact overall *P* values and adjusted *P* values are provided in Supplementary Table S4. Statistical significance was defined as *P* < 0.05. Groups sharing the same compact letter display (CLD) letter are not significantly different, whereas groups with different letters are significantly different.

Next, we examined whether DMSO exposure influenced locomotor activity. During the light phase, locomotor activity remained largely unchanged, except under specific conditions (Figures 3A–D). In contrast, during the dark phase, DMSO dose-dependently increased locomotor activity across multiple experimental groups.

To determine whether the increased nighttime locomotor activity simply reflected prolonged wakefulness immediately after lights-off, we performed an additional analysis of waking activity (beam crossings per waking minute) using data collected during ZT13–24. Waking activity was not significantly affected by dietary DMSO in young flies of either sex. In contrast, aged flies exhibited a concentration-dependent increase in waking activity, with significant increases observed at higher DMSO concentrations, particularly in 1% DMSO-treated males and in 1%–2% DMSO-treated females (Figures 3E,F).

These results indicate that DMSO broadly perturbs sleep-related behavioral organization. In aged flies, however, DMSO also increased waking activity, indicating that the increased nighttime locomotor activity cannot be explained solely by prolonged wakefulness.

### 3.4 High-dose DMSO induces systemic physiological impairment in *Drosophila*

Next, we performed survival assays under sucrose/agar conditions to evaluate whether DMSO-induced behavioral alterations were associated with broader physiological consequences.

**FIGURE 4:**
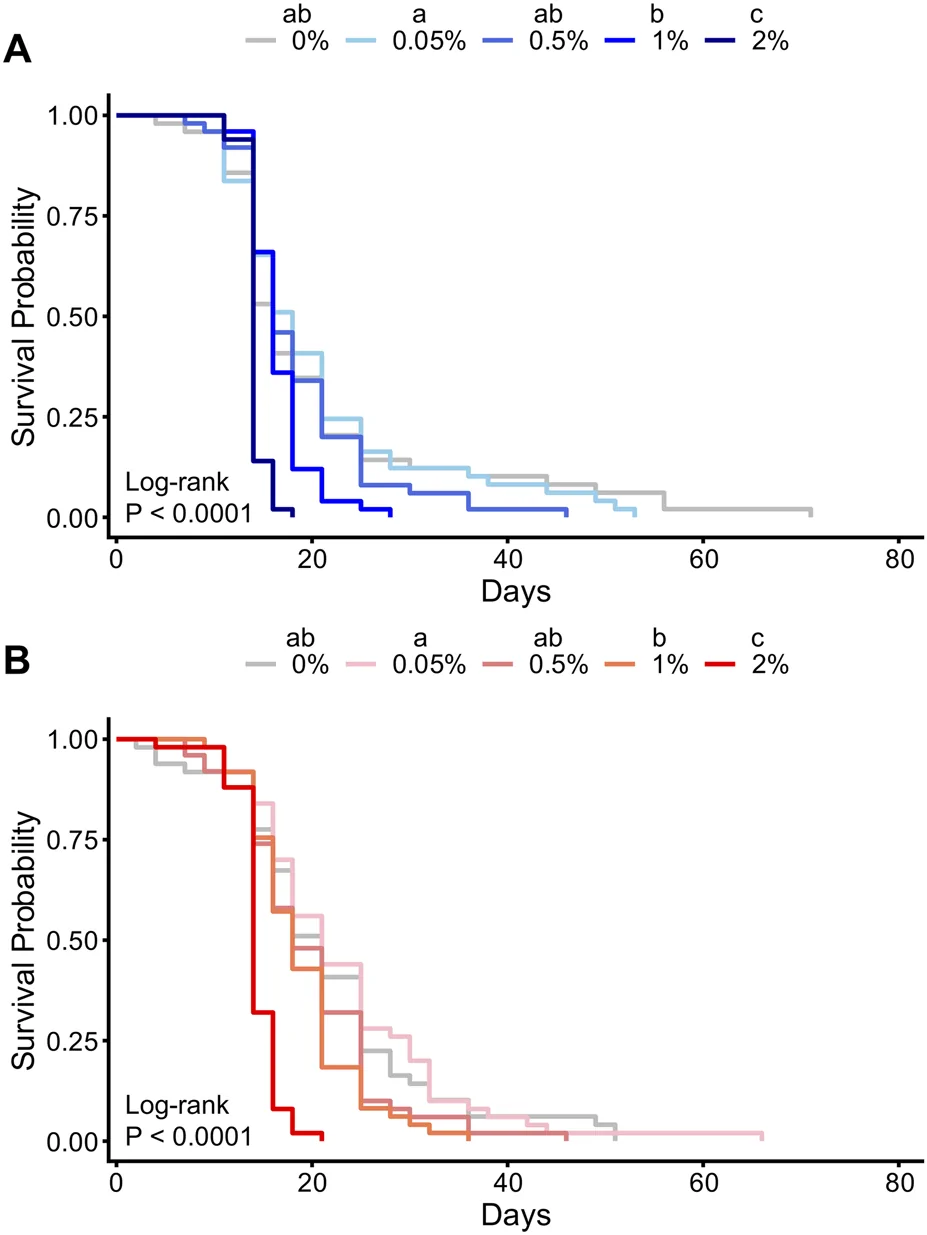
Dose-dependent effects of dietary DMSO on survival of *Drosophila* **(A,B)** Kaplan–Meier survival curves showing the effects of dietary DMSO on survival under sucrose/agar conditions. **(A)** Survival curves for males. **(B)** Survival curves for females. Flies were maintained on 2% agar supplemented with 4% sucrose containing 0%, 0.05%, 0.5%, 1%, or 2% DMSO, as indicated by the color gradient. Each group consisted of 50 flies (5 biological replicate vials, 10 flies per vial). Survival probability is plotted as a function of age (days). Overall group differences were assessed using the log-rank test (A: *P* = 1.0 × 10^−8^; **(B)** *P* = 2.0 × 10^−14^). Pairwise comparisons were performed using the log-rank test with Benjamini–Hochberg correction. Only the 2% DMSO group differed significantly from the 0% control (A: adjusted P = 7.6 × 10^−5^; **(B)** adjusted *P* = 1.5 × 10^−9^). Groups sharing the same compact letter display (CLD) letter are not significantly different, whereas groups with different letters differ significantly.

Continuous exposure to 2% DMSO significantly reduced survival in both male and female flies, whereas lower DMSO concentrations had relatively limited effects (Figures 4A,B). In the 2% DMSO group, survival rates rapidly declined beginning at approximately days 11–14, with nearly complete mortality observed by days 18–21.

These findings indicate that high-dose DMSO induces systemic physiological impairment in *Drosophila* and suggest that DMSO-induced behavioral phenotypes may occur along a continuum of toxicophysiological perturbations.

### 3.5 DMSO alters sleep-related behaviors in *Hydra vulgaris*

To determine whether DMSO also affects sleep-related behaviors in another model organism, we next examined sleep-related behaviors in one of the cnidarians, *H. vulgaris*.

To minimize the inclusion of animals exhibiting extremely low spontaneous activity, predefined FOM-based quality-control criteria were applied prior to behavioral analyses (FOM < 0.1 or > 0.9). The distributions of Day 1 and Day 2 FOM values for all animals are shown in Supplementary Figure S2. Although the proportion of excluded animals increased with increasing DMSO concentration, eight of the eighteen animals in the 2% DMSO group satisfied the predefined activity criteria and were therefore retained for analysis.

**FIGURE 5:**
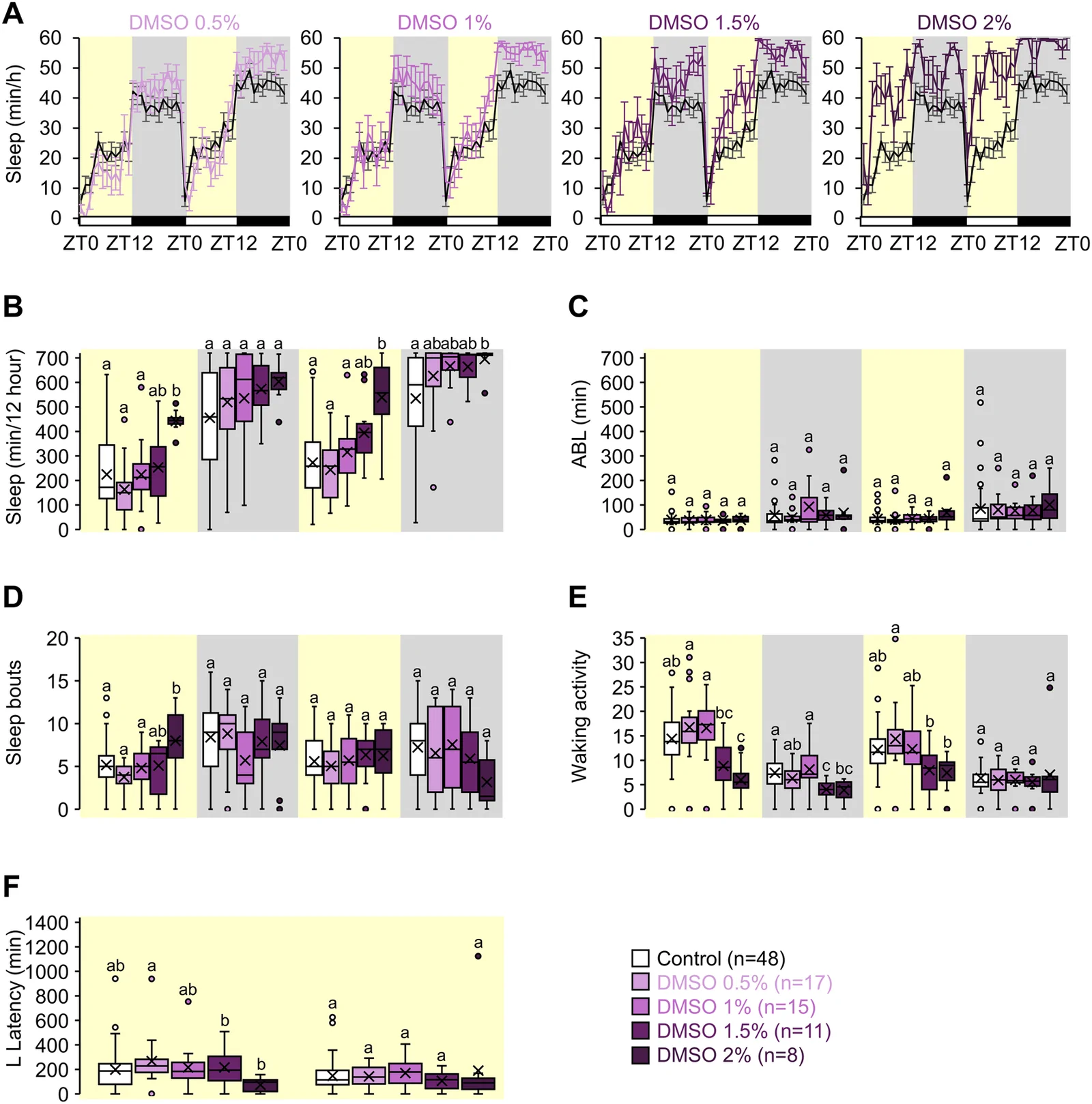
Effects of DMSO on sleep-related behavioral parameters in *Hydra*. **(A)** Sleep patterns of *Hydra* at different concentrations of DMSO. The black line represents the control group (no DMSO), and colored lines represent groups treated with DMSO at different concentrations. **(B)** Box plots representing total sleep amount during light (L) and dark (D) phases over 2 days. **(C)** Box plots representing ABL over 2 days. **(D)** Box plots representing sleep-bout number over 2 days. **(E)** Box plots representing waking activity over 2 days. **(F)** Box plots representing sleep latency over 2 days. n.s., not significant; *P < 0.05, **P < 0.01, ***P < 0.001, ****P < 0.0001 indicate significant differences between groups based on *post hoc* multiple comparisons. Sample sizes (n) are indicated in the figure. Statistical analyses were performed as follows: Kruskal–Wallis test for (**(A)** sleep Day1 night), (**(B)** ABL Day1 day and night, Day2 day and night), (**(C)** bout Day2 night), (**(D)** waking activity Day2 night), and (**(E)** latency Day2); Kruskal–Wallis test followed by Dunn’s multiple-comparison test with Holm correction for (**(A)** sleep Day1 day and Day2 night), (**(C)** bout Day1 day), (**(D)** waking activity Day1 day and Day2 day), and (**(E)** latency Day1); one-way ANOVA for (**(C)** bout Day1 night and Day2 day); one-way ANOVA followed by Tukey’s test for (**(A)** sleep Day2 day); and Welch ANOVA followed by Games–Howell test for (**(D)** waking activity Day1 night). Groups sharing the same compact letter display (CLD) letter are not significantly different, whereas groups with different letters differ significantly. Exact overall *P* values and adjusted *P* values are provided in Supplementary Table S5.

No major changes in sleep-related parameters were detected in the 0.5% DMSO group (Figures 5A–F). However, 2% DMSO significantly altered sleep-like behaviors. Specifically, total sleep amount increased with an increase in sleep-bout number, whereas average bout length remained largely unchanged (Figures 5B–D). Waking activity and sleep latency were reduced under 2% DMSO conditions (Figures 5E,F). Dark-phase sleep latency was not analyzed because *Hydra* does not exhibit a synchronized behavioral response to lights-off, precluding reliable measurement of sleep latency after the onset of darkness. Importantly, no overt morphological abnormalities were observed throughout the behavioral recording period at this concentration (Supplementary Figure S3).

In contrast, higher concentrations (3% and 5%) of DMSO caused severe morphological deterioration and death before completion of the recording period, precluding behavioral analyses and indicating profound physiological disruption (Supplementary Figure S3).

Although the direction of total sleep changes differed between *Drosophila* and *Hydra*, DMSO increased sleep-bout number in *Drosophila*, whereas in *Hydra* this effect was observed only under the 2% DMSO condition during the first light phase. These findings suggest that DMSO perturbs sleep-related behavioral organization in both species, although the resulting physiological and behavioral outcomes are species dependent.

## 4 Discussion

### 4.1 DMSO is not an inert vehicle in sleep research

In this study, we demonstrated that DMSO significantly perturbed sleep-related behavioral phenotypes in both *Drosophila* and *Hydra*. In *D*. *melanogaster*, DMSO altered nighttime sleep behavior, increased sleep-bout number, reduced average sleep-bout length, and elevated locomotor activity. In *H*. *vulgaris*, DMSO altered sleep-like behaviors including increased sleep-bout numbers and reduced waking locomotor activity. At high concentrations, DMSO induced severe physiological impairment including shortened lifespan in *Drosophila* and morphological deterioration and death in *Hydra*. Together, these findings demonstrate that DMSO is not merely a passive experimental vehicle but a biologically active perturbant that can substantially influence behavioral phenotypes.

Importantly, most of the observed effects occurred within the concentration ranges commonly used in pharmacological and behavioral studies. Although DMSO is generally assumed to be biologically inert at low concentrations, our results demonstrate that even moderate DMSO exposure can alter sleep-related behavioral organization. These findings raise important concerns regarding the interpretation of sleep-related behavioral phenotypes in experiments using DMSO as a vehicle control. Accordingly, we recommend that future pharmacological and behavioral studies using DMSO as a vehicle include matched DMSO-only control groups. Such controls will help distinguish sleep-related behavioral effects attributable to DMSO itself from those caused by the experimental compounds.

### 4.2 Sleep architecture is highly vulnerable to physiological perturbation

Notably, DMSO consistently altered the sleep-bout organization in both species. In *Drosophila*, DMSO reduced the average sleep-bout length while increasing sleep-bout number, indicating fragmentation of sleep architecture. In *Hydra*, the total amount of sleep-like behavior increased following exposure to 2% DMSO; however, sleep-bout number also increased. Although the direction of total sleep differed between the species, both organisms exhibited destabilized sleep-related behavioral organization following DMSO exposure.

Notably, in *Hydra*, sleep-related behavioral alterations were detected under 2% DMSO despite the absence of overt morphological abnormalities throughout the behavioral recording period. This observation suggests that sleep-related behavioral organization may represent a sensitive indicator of physiological perturbation before overt morphological abnormalities become apparent.

These observations suggest that sleep architecture is particularly sensitive to subtle physiological disturbances. Consistent with this interpretation, analysis of waking activity showed that the increased nighttime locomotor activity observed in aged *Drosophila* could not be explained solely by prolonged wakefulness, indicating that DMSO also increased locomotor activity during wakefulness in aged flies. Unlike total sleep duration alone, parameters such as sleep-bout stability, fragmentation, and sleep latency reflect the integrated state of multiple physiological systems. Sleep emerges from complex interactions among neuronal excitability, metabolism, oxidative stress, membrane physiology, and homeostatic regulation (Saper et al., 2010; Sehgal and Mignot, 2011; Joiner, 2016). Consequently, relatively nonspecific physiological perturbations induced by solvents may be amplified as measurable changes in sleep-related behavioral organization.

This vulnerability may be particularly important in sleep research because sleep-related behavioral phenotypes are often interpreted as evidence of specific molecular or neuronal mechanisms. Our findings suggest that some behavioral phenotypes observed in pharmacological experiments may instead reflect relatively broad solvent-induced physiological perturbations rather than direct modulation of sleep-regulatory pathways.

### 4.3 Species-dependent behavioral outcomes complicate interpretation of solvent effects

An important aspect of the present study is the substantial species-dependent variability in behavioral outcomes. In *Drosophila*, DMSO predominantly reduced nighttime sleep and increased locomotor activity, whereas in *Hydra*, 2% DMSO increased total sleep-like behavior while reducing waking locomotion. These contrasting outcomes indicate that DMSO does not exert a simple unidirectional effect on sleep-like behaviors.

Instead, the behavioral consequences of DMSO application probably depend on species-specific physiological properties including the organization of the nervous system, membrane composition, metabolic state, and stress-response pathways. In addition to intrinsic physiological differences, the route of DMSO administration may also contribute to the species-dependent behavioral outcomes observed in this study. In *Hydra*, DMSO is absorbed directly through the body surface from the surrounding medium, whereas in *Drosophila* it is administered orally through the food. Consequently, the behavioral effects observed in *Drosophila* may reflect not only direct actions on the nervous system but also indirect effects mediated through the digestive system. Although direct evidence that DMSO alters gut physiology in *Drosophila* is currently lacking, differences in the route of exposure may influence the physiological targets and thereby contribute to the distinct behavioral outcomes observed between species. Such variability complicates the interpretation of solvent-induced phenotypes because similar physiological perturbations may manifest differently in different organisms.

Importantly, despite these species-dependent differences in total sleep amount, increased sleep-bout numbers were observed in both organisms. Although this common behavioral feature suggests that sleep architecture is sensitive to DMSO exposure in both species, the distinct phenotypic outcomes indicate that the underlying mechanisms and behavioral consequences are likely species dependent.

### 4.4 High-dose DMSO induces systemic toxicophysiological disruption

At high concentrations, DMSO induced severe systemic physiological impairments in both species. Continuous exposure to 2% DMSO markedly shortened the lifespan of *Drosophila*, whereas concentrations of 3%–5% caused profound morphological deterioration and death in *Hydra*. These findings are consistent with previous reports describing developmental toxicity, reduced survival, and physiological dysfunction induced by high concentrations of DMSO in multiple organisms (Cvetković et al., 2015; AlOkda and Van Raamsdonk, 2022; Gomes et al., 2025; Thi Thu Trinh et al., 2025).

Importantly, our results suggest that solvent-induced behavioral phenotypes and overt toxicity may exist along a continuous toxicophysiological spectrum rather than representing entirely separate phenomena. Therefore, behavioral alterations observed at relatively low DMSO concentrations may reflect early or mild physiological perturbations that become progressively more severe at higher doses.

Notably, no overt morphological abnormalities were observed throughout the behavioral recording period in the 2% DMSO-treated *Hydra*, despite significant alterations in sleep-related behavioral parameters. In contrast, exposure to 3% and 5% DMSO caused severe morphological deterioration before completion of the recording period, precluding behavioral analyses. These observations indicate that behavioral alterations may become detectable before overt morphological abnormalities are apparent.

Nevertheless, because 2% DMSO lies close to concentrations that produced overt physiological deterioration, we cannot completely exclude the possibility that some of the observed behavioral changes reflect early physiological impairment in addition to altered sleep regulation. Furthermore, although only animals satisfying the predefined FOM-based quality-control criteria were included in the behavioral analyses (Supplementary Figure S2), the retained animals in the 2% DMSO group exhibited lower FOM values than those in the control group, resulting in a shift of the FOM distribution toward lower values. Therefore, subtle physiological changes that were not sufficient to exclude the animals from analysis may nevertheless have contributed to the reduced locomotor activity and increased sleep-like immobility. Accordingly, the behavioral quiescence observed in *Hydra* at this concentration should be interpreted as “sleep-like immobility” rather than a definitive “sleep-like state”, and these findings should be interpreted with appropriate caution. Similarly, because behavioral analyses in *Drosophila* included only flies that remained viable until the end of the recording period, survivorship bias cannot be completely excluded, particularly in the 2% DMSO group of aged flies. Accordingly, the behavioral phenotypes observed at the highest DMSO concentration should be interpreted with this potential limitation in mind.

### 4.5 Potential mechanisms underlying DMSO-induced perturbation of sleep-related behaviors

The present study primarily focused on behavioral phenotypes; however, several mechanisms may have contributed to the effects of DMSO. Because none of these mechanisms were directly examined in the present study, the following discussion should be regarded as speculative and intended only to provide possible biological interpretations of our behavioral observations. One possible mechanism involves alterations in membrane properties. Previous studies have shown that DMSO can modify the organization of the lipid bilayer, membrane fluidity, and membrane permeability, which may broadly influence neuronal excitability and cellular homeostasis (Notman et al., 2006).

Another possible explanation involves oxidative stress. Previous studies have reported that DMSO can increase intracellular reactive oxygen species (ROS) levels, particularly at high concentrations. Because oxidative stress is closely associated with sleep quality, sleep fragmentation, and physiological stress responses, accumulation of reactive oxygen species may contribute to destabilization of sleep-related behavioral organization observed in the present study (Koh et al., 2006; Hill et al., 2018; Sangweni et al., 2021).

In addition, DMSO has been reported to inhibit acetylcholinesterase activity, potentially enhancing cholinergic signaling (Kumar and Darreh-Shori, 2017). In *Drosophila*, cholinergic pathways play important roles in arousal regulation, raising the possibility that altered cholinergic signaling may contribute to increased locomotor activity and reduced nighttime sleep observed under DMSO exposure (Yi et al., 2013).

Interestingly, the apparent delay in the evening activity peak observed in young flies exposed to 2% DMSO raises the possibility that high concentrations of DMSO may influence circadian timing in addition to sleep regulation. Although the present study was not designed to examine circadian clock function directly, the shift in evening activity suggests that DMSO may affect circadian output or clock-controlled behaviors. Future studies will be required to determine whether DMSO directly influences the circadian oscillator or primarily alters downstream behavioral outputs.

However, given the broad physicochemical effects of DMSO, the observed phenotypes are unlikely to originate from a single mechanism. Rather, the observed phenotypes may reflect multiple simultaneous physiological perturbations induced by DMSO, although the relative contribution of each mechanism remains unknown. Further studies will be required to determine which of these mechanisms contributes most strongly to the behavioral phenotypes observed under different experimental conditions and in different species.

### 4.6 Implications for pharmacological and behavioral studies

Our findings emphasize the importance of carefully evaluating solvent effects in behavioral and sleep research. Because sleep-related behavioral phenotypes are highly sensitive to physiological perturbations, solvent-induced effects may substantially confound the interpretation of pharmacological experiments, particularly when subtle behavioral phenotypes are analyzed.

Importantly, in the present study, DMSO was used as a representative example of common solvents. However, the broad implications of our findings extend beyond DMSO itself. Vehicle solvents should not automatically be regarded as biologically inert controls, specifically in studies investigating highly integrative physiological phenomena such as sleep.

Therefore, future studies should carefully validate solvent concentrations, assess potential solvent-induced behavioral effects independently, and interpret pharmacological phenotypes with caution. Collectively, our results further suggest that analysis of sleep architecture and behavioral organization may provide sensitive indicators of solvent-induced physiological perturbations before overt toxicity becomes apparent.

## Data Availability

The original contributions presented in the study are included in the article/Supplementary Material, further inquiries can be directed to the corresponding author.
