# Supplementary material for: Dimethyl sulfoxide perturbs sleep-related behavioral organization in multiple species and confounds interpretation of sleep phenotypes
Source: Front Toxicol. 2026 Aug 21;8:1907359. doi: 10.3389/ftox.2026.1907359 (PMC13541398; doi:10.3389/ftox.2026.1907359)
Supplement: Supplementary file 1 [file DataSheet1.pdf]

Supplementary Material

Dimethyl sulfoxide perturbs sleep-related behavioral organization in multiple species and confounds interpretation of sleep phenotypes

Nanami Udo [1†], Manabu Sekiguchi [2†], Etsuko Sawatari [2], Taichi Q. Itoh [2*]

[1] Graduate School of Systems Life Sciences, Kyushu University, Fukuoka, Japan.

[2] Faculty of Arts and Science, Kyushu University, Fukuoka, Japan.

†These two authors contributed equally to this work

*** Correspondence:**
Taichi Q. Itoh
taichi.itoh@kyudai.jp

Keywords: DMSO, *Drosophila melanogaster*, *Hydra vulgaris*, Sleep architecture, Behavioral phenotyping, Sleep fragmentation, Toxicophysiology.

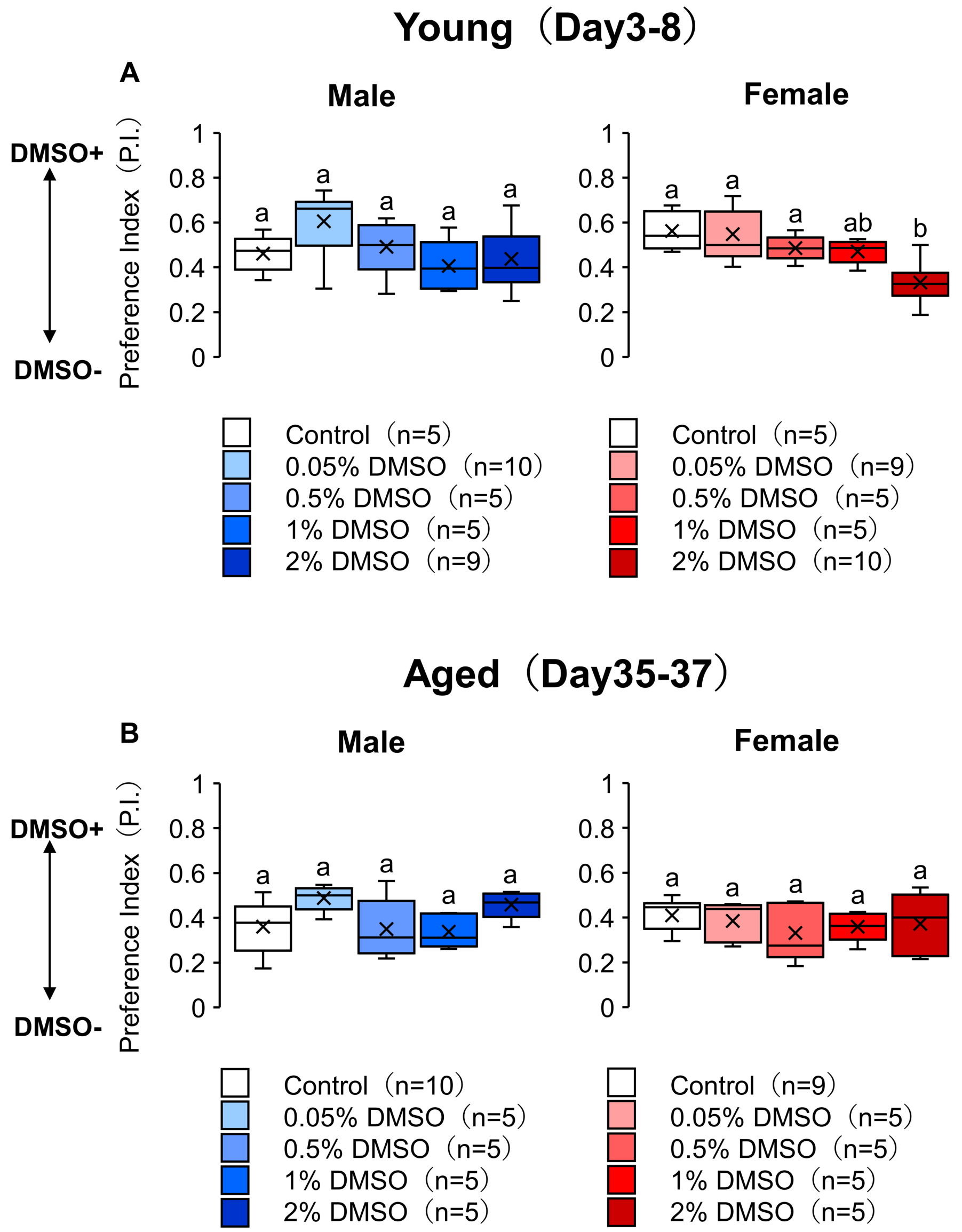

Supplementary Figure S1. Two-choice preference assay for dietary DMSO in young and aged flies.

(A) Young and (B) aged flies were subjected to a two-choice preference assay comparing a sucrose solution containing DMSO (DMSO+) with a sucrose-only control solution (DMSO−). Preference was quantified as a Preference Index (PI), where PI = 1 indicates complete preference for the DMSO-containing solution and PI = 0 indicates complete avoidance (complete preference for the control solution). Male (blue) and female (red) data are shown separately. Color intensity denotes DMSO concentration (Control, 0.05%, 0.5%, 1%, and 2%). Box plots indicate the median (center line) and interquartile range (box), with whiskers indicating the data range; crosses indicate the mean. Sample sizes (n) for each group are shown in the figure. Statistical significance was defined as $P < 0.05$. Groups sharing the same compact letter display (CLD) letter are not significantly different, whereas groups with different letters differ significantly. Exact overall P values and Holm-adjusted pairwise P values are provided in Supplementary Table S1.

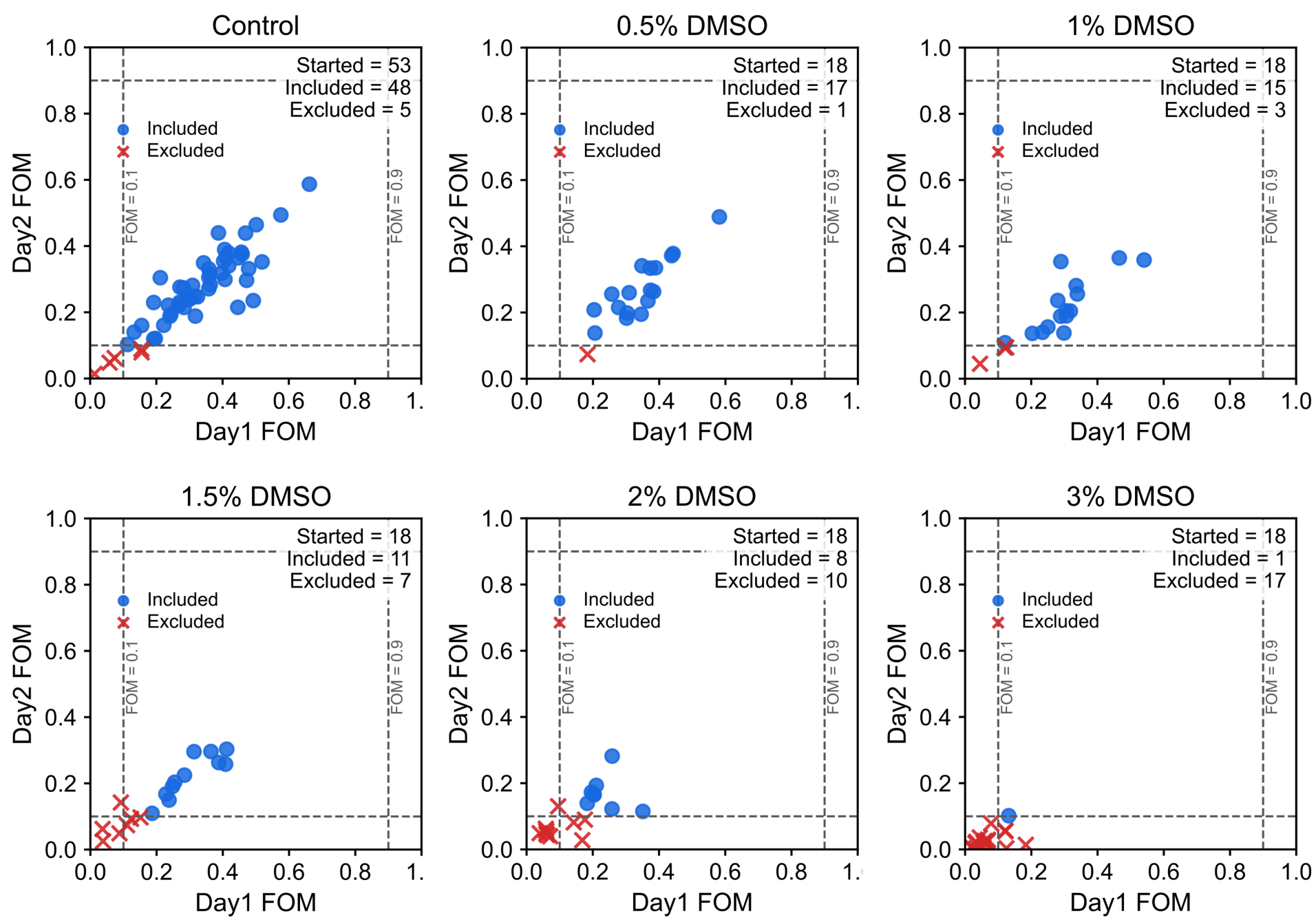

Supplementary Figure S2. Distribution of Fraction of Movement (FOM) values used for quality control.

Each point represents one *Hydra*. Animals with mean FOM values between 0.1 and 0.9 on both Day 1 and Day 2 were included in the behavioral analyses (blue circles), whereas those outside these predefined thresholds were excluded (red crosses). Dashed lines indicate the FOM thresholds used for quality control.

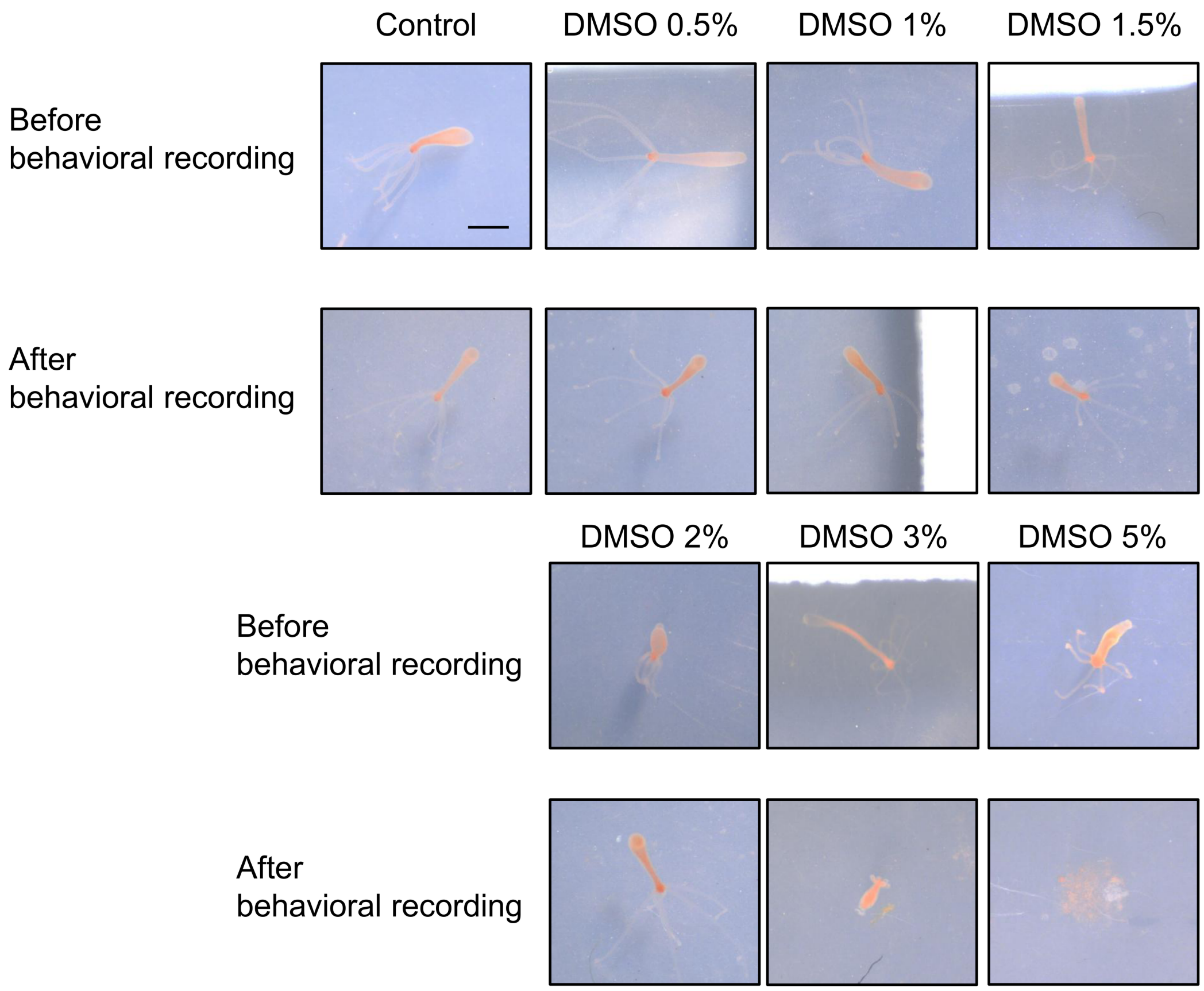

Supplementary Figure S3. Effects of different DMSO concentrations on Hydra morphology.

Representative images of *Hydra vulgaris* before and after behavioral recording under the indicated DMSO concentrations. Black bars indicate 1 mm.
